# CD28 Superagonist Shock and Blockage of Motogenic T Cell Cascade

**DOI:** 10.3389/fimmu.2021.670864

**Published:** 2021-04-21

**Authors:** Karl-Gösta Sundqvist

**Affiliations:** Department of Laboratory Medicine, Division of Clinical Immunology, Karolinska Institute and Clinical Immunology and Transfusion Medicine Karolinska University Hospital, Stockholm, Sweden

**Keywords:** costimulation, motiity, adhesion, activation, CD28

## Introduction

T cell motility is arrested by T cell receptor (TCR) recognition of cognate peptide-MHC (pMHC) complexes on antigen-presenting cells (1). Co-stimulation through CD28 is required for immune responses but its influence on T cell motility is less clear than that of the TCR. Ligation of CD28 and TCR collaborate to block a distinct protease-controlled step in the motogenic T cell cascade directed by the large transmembrane receptor low density lipoprotein receptor-related protein 1 (LRP1) and its high molecular weight ligand thrombospondin-1 (TSP-1) (2–9). Here I describe how blockage of this protease step is a likely explanation for the instantaneous arrest and cytokine storm elicited by the CD28 superagonist TGN1412 in a clinical trial in 2006 (10), which illustrates the power of the LRP1-targeted protease-dependent T cell control.

## LRP1- and TSP-1-Directed Motogenic Cascade

LRP-1 and its coreceptor calreticulin trigger T cell motility and integrin-dependent adhesive contacts through a crosslinking cascade *via* the NH-terminal region of TSP-1 and interaction of the COOH-terminal region with CD47 (2–9). LRP1 and TSP-1 have a high turnover, their expression depends on sensing of components of the extracellular matrix (ECM) and other cells and they promote motility in response to ligation of integrin and chemokine receptors. Shedding of LRP1, which is attributed to a disintegrin and metalloprotease 10 (ADAM10), prevents accumulation of LRP1 and TSP-1 on the cells, and hence integrin-dependent adhesion and T cell activation, while maintaining polar cell shape and motility (9). Ligation of integrin and chemokine receptors antagonizes shedding through stimulation of transport of TSP-1 to the cell surface, whereupon TSP-1 targets shedding (5, 9). However, unlike a broad-spectrum MMP inhibitor, ligation of integrin or chemokine receptors by their natural ligands merely reduces but does not abrogate shedding.

## Ligation of TCR/CD3 and CD28 Targets LRP1 and TSP-1

The LRP1- and TSP-1-directed sensing and motility mechanism probably plays a crucial role for T cell search and surveillance to bring TCR and CD28 in contact with cognate pMHC complexes and B7. It is therefore interesting that these interactions target LRP1 and TSP-1 (2–9). Ligation of CD28 antagonizes shedding of LRP1 directly, whereas ligation of TCR/CD3 in collaboration with integrin ligands increases TSP-1 on cells and TSP-1 inhibits shedding of LRP1 (4, 5, 9). This indicates that ligation of the TCR/CD3 complex and CD28 collaborate to prevent shedding of LRP1. Ligation of TCR/CD3 in a memory T cell clone enhanced cell surface expression of LRP1 suggesting a direct effect on shedding (4) but an impact on CD28 of the antigen-presenting cells used to maintain this clone cannot be excluded.

T cell activation by antigen upregulates TSP-1 synthesis and down-regulates LRP1 synthesis (8). The expression of LRP1 and TSP-1 in activated cells requires CD28 co-stimulation, since blocking co-stimulation by soluble CTLA-4 virtually abolishes expression of TSP-1 and LRP1 (8). The decreased LRP1 synthesis probably contributes to reduce motility, whereas the antigen-induced potentiation of TSP-1 expression, in collaboration with LRP1, supports integrin-dependent adhesive contacts with antigen-presenting cells.

Besides the initial effects of antigen stimulation on LRP1 and TSP1, antigen-induced IL-2 may stimulate TSP-1 synthesis contributing to subsequent effects on motility and perhaps also on other cells through secreted TSP-1 (6).

## CD28 Superagonists and Blockage of Counter-Adhesive Step of the Motogenic T Cell Cascade

A single dose of the superagonistic CD28 antibody TGN1412 induced cytokine release syndrome in healthy human volunteers (10). This reflects capacity to crosslink CD28, binding to Fc-receptors, and activation of effector memory cells (11–14). Preculture at high cell density also increases the sensitivity of T cells to activation by antigen and TGN1412. This is thought to reflect tonic signaling (12), which is further discussed below.

Rodent experiments show that the primary effect of superagonistic CD28 antibodies is instantaneous arrest and trapping of T cells in lymphoid tissues associated with irregular cell shape, increased cell size and activation (15). This is accompanied by increased capacity to bind to integrin ligands and most likely reflects adhesion and cytoplasmic spreading, and probably caused the lymphopenia developed in the clinical trial (10). The TGN1412 trial and accompanying studies hence show that ligation of CD28 targets T cell motility and adhesion, like ligation of the TCR, which induces polarizing interactions with antigen-presenting cells (1, 16) contrasting with the apolar adhesion by CD28 superagonists (15). These similar effects probably require a mechanism targeted *via* both TCR and CD28, a criterion fulfilled by the motogenic cascade directed by LRP1 and TSP-1) (2–9).

**Figure 1:**
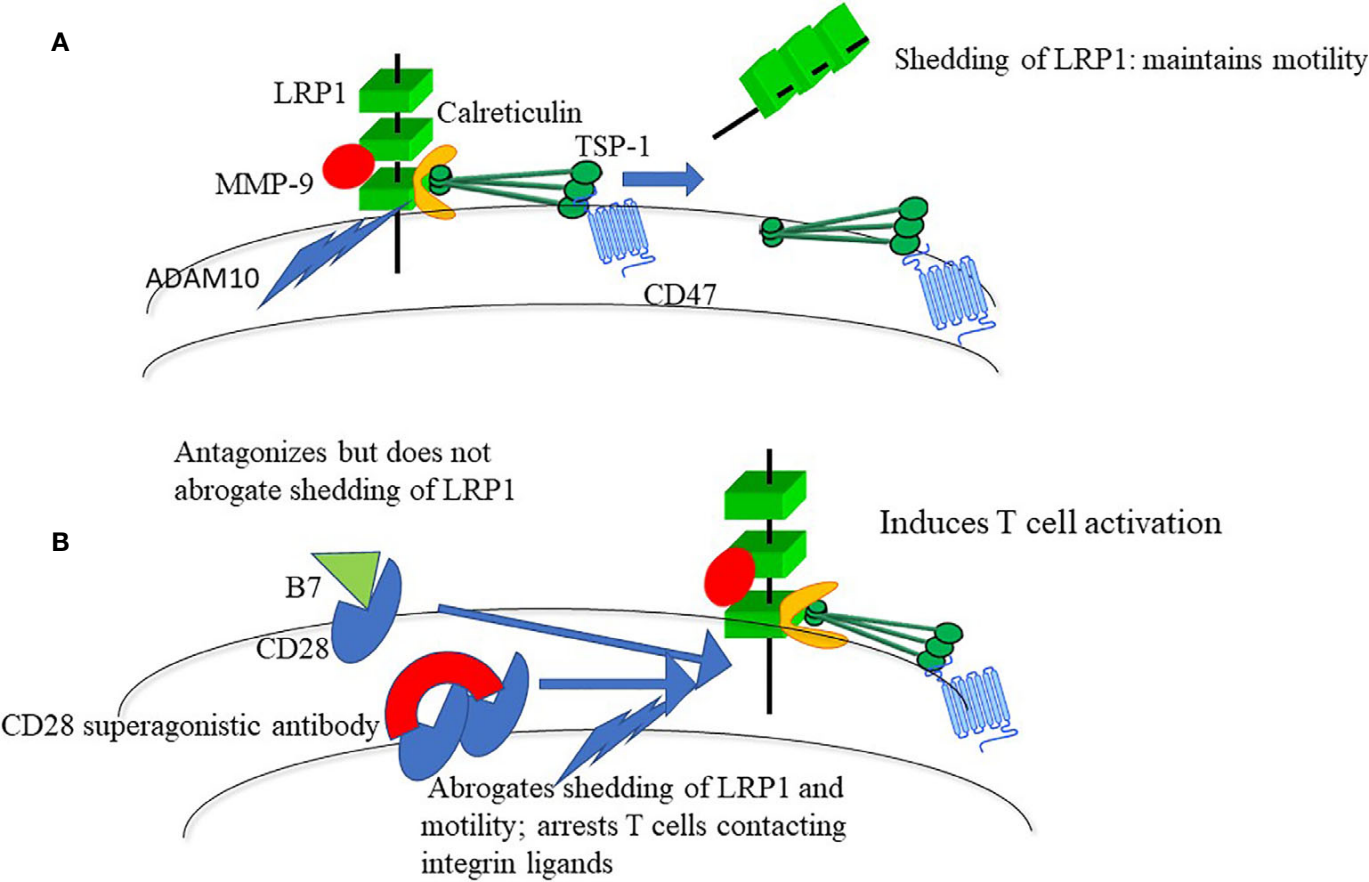
**(A)** LRP1-calreticulin complexes trigger motility through the NH-terminal domain of TSP-1 and a coupled interaction of the COOH-terminal with CD47. Shedding of LRP1, prevents persistent integrin-mediated adhesion. T cells are hence programmed to move during contact with integrin ligands. LRP1 interacts with more than 40 ligands and shedding may act to control such interactions. One such interaction, to MMP9, is shown since it is a ligand for LRP1 (17) and probably is important for T cell motility (18). **(B)** Interaction of B7 with CD28 antagonizes shedding of LRP1, whereas CD28 superagonists, which crosslink CD28, abrogate shedding of LRP1.

A clue to the mechanism behind the disastrous TGN1412 trial is that ligation of CD28 antagonizes shedding of LRP1 (**Figure 1**) (2, 9). Ligation of CD28 using a conventional CD28 antibody has a moderate inhibitory effect on shedding of LRP1, whereas broad-spectrum MMP inhibition abrogates shedding. This induces adhesion and apolar spreading of T cells contacting integrin ligands, and a characteristic irregular periphery with pseudopodia (2, 9), which mimics the appearance of arrested cells in rodents treated with superagonistic CD28 antibodies (15). Like TGN1412, broad-spectrum MMP inhibition promotes excessive T cell production of cytokines and, in addition, of chemokines and growth factors (19). This reinforces the conclusion that the CD28-targeted protease of the motogenic LRP1-TSP-1 cascade controls T cell adhesion and activation.

## The Motogenic LRP1-TSP-1 Cascade and Tonic Signaling

Constitutive low level signaling, often called tonic, is thought to drive T cell motility and responsiveness to antigen and TGN1412 through TCR self MHC interaction (12, 20–22). The conditions promoting tonic signaling and responsiveness to antigen and TGN1412 have several similarities to the motogenic cascade suggesting a shared mechanistic background. Maintenance of motility through the motogenic cascade hence also depends on contact with other cells but also on contact with ECM (2, 23–25). Additional evidence for a shared background of the tonic signaling responsible for the action of TGN1412 and the motogenic cascade is that absence of cell contact causes loss of sensitivity to TGN1412 and antigen in blood T cells (12), which resembles that T cell motility is low in blood and high in lymphoid tissue (26).

Motility dependent on sensing of ECM components and other cells (23–25) by the motogenic LRP1-TSP-1-directed cascade (2) implies a high degree of adaptivity to different environments in comparison with motility driven by self-recognition. In tissues, T cells frequently encounter ECM components, such as collagen and fibronectin, besides other cells, and it is therefore logical that contact both with cellular and non-cellular components supports T cell motility. In support of a basal motility mechanism dependent on environmental sensing, cell contact supports protein synthesis (27) and protein synthesis is required for motility of amebae and lymphocytes (24, 28–30).

## OKT3-Induced Cytokine Release Syndrome

OKT3 induces self-limited cytokine release and spontaneously reversible clinical symptoms (31), similar to but less pronounced than the TGN1412-induced life-threatening stereotypic symptoms in all individuals (10). These TCR/CD3-mediated effects, together with the fact that OKT3 induces T cell arrest (32), therefore resemble the effects of broad spectrum MMP inhibition on motility and cytokine release. This indicates that OKT3 targets the protease step of the LRP1- and TSP-1-directed motogenic cascade. OKT3 can activate T cells *in vitro* without preculture at high density, unlike TGN1412, which probably reflects independence of accessory cells and tonic signaling (12). This probably means that the cells do not have to be motile to bind OKT3, in contrast to TGN1412, which is presented bound to environmental Fc receptors.

## LRP1 and TSP-1 Control of Cell Signaling

LRP1 and TSP-1 may regulate T cell functions, such as adhesion and activation, by connecting cell surface receptors and signaling pathways. LRP1 directs T cell motility through JAK tyrosine kinase and PI3K (3) and evidence from non-lymphoid cells indicate that LRP1 is a coreceptor for other receptors and an integrator of signaling pathways through its more than 40 binding sites for other molecules (33–36). One example is the influence of LRP1 on glucose metabolism (37), which is crucial for T cell activation and requires CD28 (38). Additional examples of integration of signaling pathways is that LRP1 is a receptor for TGF-β, acts in concert with other TGF-β receptor types (39), and together with TSP-1 (40) control TGF-β action, which may be important since TGF-β has a crucial role for T cell function with far-reaching implications for Th17 and Treg cells (41).

## Conclusions and Perspectives

Encounter of the TCR and CD28 with cognate pMHC complexes and B7 on antigen-presenting cells hence seems to block a protease of a molecular cascade regulating T cell motility and integrin-dependent contacts. The powerful effects of broad-spectrum MMP inhibition on T cell motility and activation and the similarities of these effects with the TGN1412- induced cytokine storm indicate that this protease control is important. This control seems to maintain cell surface expression of LRP1 and associated ligands at a low level, enough for triggering motogenic signals but insufficient for integrin-dependent adhesive contacts and activation. The low level of stable LRP1 expression on the cells due to shedding probably prevents LRP1, by virtue of its multiple binding sites for other molecules (33–36), to connect with them and signaling pathways critical for activation.

Unlike broad-spectrum MMP inhibition and superagonistic CD28 antibodies, which both may effectively inhibit the protease controlling cell surface expression of LRP1 to abrogate shedding, pMHC complexes and B7 seem to collaborate to inhibit this protease. The putative existence of this control with a driving effect on motility is evident in T cells with upregulated integrins in an environment presenting the corresponding integrin ligands (42). In addition, this control may be of vital importance to prevent triggering of adverse immune responses, which indeed is demonstrated by the cytokine release syndrome induced by superagonistic CD28 antibodies. So, the counteradhesive motogenic protease mechanism may function as an important checkpoint, decisive for the behavior and fate of T cells, coupled to the encounter of the TCR and CD28 with pMHC complexes and B7. This protease mechanism may further prevent the large LRP1 molecule from interference with TCR-pMHC interactions and generate force on TCR-pMHC bonds in T cell antigen recognition, which is a matter of debate (43).

The dependence of the motogenic molecular cascade on sensing of the environment implies a constitutive property of crucial importance for the search for antigen and immune surveillance. The protease step endows this cascade with a fundamental control of triggering of immune responses. A basic regulation of T cell motility and adhesive contacts distinct from but responsive to the TCR and CD28 is logical from an evolutionary perspective. Adaptive immunity evolved in jawed vertebrates is hence likely to be intimately dependent on phylogenetically old proteins, including LRP1, TSP-1, and integrins, and functions dependent on them, such as motility and adhesion, evolved at earlier stages of evolution.

## Author Contributions

The author confirms being the sole contributor of this work and has approved it for publication.

## Funding

This work was supported by the Swedish Research Council and the Swedish Cancer Foundation.

## Conflict of Interest

The author declares that the research was conducted in the absence of any commercial or financial relationships that could be construed as a potential conflict of interest.
